# From static snapshots to dynamic panoramas: the evolution and future vision of palliative care atlas in cross-national perspectives

**DOI:** 10.1007/s43999-024-00043-z

**Published:** 2024-04-18

**Authors:** Vilma A. Tripodoro, Juan José Pons, Fernanda Bastos, Eduardo Garralda, Álvaro Montero, Ana Cristina Béjar, Carlos Centeno

**Affiliations:** 1 Global Observatory of Palliative Care-WHO Collaborating Centre, Institute of Culture and Society, ATLANTES, University of Navarra, Pamplona (Navarra), Spain; 2 IdiSNA, Instituto de Investigación Sanitaria de Navarra, Pamplona (Navarra), Spain; 3 Department of History, History of Art and Geography, University of Navarra, Pamplona (Navarra), Spain

**Keywords:** Atlas, Palliative care, Global health, Public health, Cartography

## Abstract

Palliative care is essential to global health services as it improves the quality of life of patients, their families and caregivers. The ATLANTES Global Observatory of Palliative Care (University of Navarra) was created a decade ago to promote a positive attitude towards patients with advanced illness in society and medicine. To do so, and over the past 15 years, ATLANTES has mapped palliative care data worldwide using public health, macro and comparative perspectives in different atlases. These have enabled data to be contextualised and good examples to be identified concisely and graphically. Atlases have been widely employed as advocacy tools within international institutions and ministries of health. While the aim and the perspective have remained unalterable over time, the methods and design have evolved throughout the publications from sober cartography and static infographics to big interactive data visualisation web tools. By embracing technology, ATLANTES has developed an open-access web mapping tool reuniting information from regional atlases, favouring global access to data. In 2022, matching the increasingly recognised need for robust monitoring of palliative care worldwide, ATLANTES became a WHO Collaborating Centre for the Global Monitoring of Palliative Care Development. This attempt to bridge the gap and ensure equitable care information in countries with limited palliative care access has resulted today in more accessible, self-explanatory, and visually appealing palliative care data.

## Introduction

Palliative care (PC) is the active, holistic care of individuals across all ages with serious health-related suffering due to severe illness, especially those near the end of life. It aims to improve the quality of life of patients, their families and their caregivers [1]. Global PC, a vital yet multifaceted component of health services, presents an intricate tapestry of development across various regions [2]. As a critical aspect of global health, its provision requires careful measurement to enhance understanding, evaluation, and, ultimately, care delivery.

The inception of the Lancaster End of Life Observatory by David Clark marked a seminal moment in the mapping of global PC. Through the concerted efforts of key local experts, it broadened our understanding across diverse cultural and national landscapes. Beginning with a detailed analysis in seven European countries [3], the observatory’s approach evolved, culminating in a comprehensive publication in 2006 that shed light on PC development across Africa [4]. This standardised template allowed for nuanced cross-country comparisons, charting the specific challenges and milestones within the continent.

The World PC Map further quantified the global progression of PC [2, 5, 6]. A classification system developed by Michael Wright in 2008 [5] and subsequent updates have underscored advancements and ongoing challenges in the PC landscape. With the collaboration of the University of Navarra, the latest recalibration in 2019 integrated ten indicators of PC provision into a developmental framework, reflecting an intertwined relationship between PC development and economic and health indicators [7]. The Quality of Death Index, a benchmarking tool from the Economist Intelligence Unit, highlighted disparities in end-of-life care while promoting the integration of PC into health systems [8]. Notwithstanding some criticism, its preference-based scoring algorithm offered a unique lens for evaluating PC quality. Human Rights Watch has championed PC as a fundamental human right, revealing the dire needs unmet in global pain treatment and PC and advocating for the accessibility of essential medicines [9].

Essentially, a PC Atlas is a monograph that compiles the latest information on the various aspects of PC development across every nation in a region. The data chiefly comes from a dedicated study exploring national outcomes based on various health indicators. The ATLANTES Global Observatory of PC, based in the University of Navarra, has provided an invaluable visual depiction of PC development through its PC Atlas Project. These atlases have become instrumental in informing policies and enhancing PC services internationally [10–12].

This article embarks on a chronological journey, examining pivotal contributions to the field and the emergent significance of the atlases in this domain. Furthermore, it will delve into the evolution, significance, impact and future vision of the PC Atlas Project, reflecting on the dynamic panorama of PC from cross-national perspectives.

### The development of the ATLANTES global observatory of palliative care

ATLANTES, launched ten years ago, is a multidisciplinary research group that aims to promote a positive attitude in society and medicine toward the care of patients with advanced and irreversible illnesses. It focuses on the problem primarily from the social sciences and humanities perspective. Its research and dissemination activities provide the various social actors with a vision based on human dignity and professional care.

The PC atlases were conceived over twenty years ago at the University of Navarra as an innovative, graphic, and succinct method of presenting PC data for a world region. This concept was inspired by one of the authors who, during the early years of PC, created a map of palliative care in Spain to synthesise a directory of services survey. This map was widely reproduced at conferences and in publications, and many palliative care teams and services proudly displayed it on the walls of their offices in hospitals as a symbol of a thriving reality. This innovative way to show this information inspired a graphic and concise method for presenting PC data globally.

ATLANTES became the WHO Collaborating Centre for Global PC Monitoring in 2022. The WHO framework of PC highlights the need for robust monitoring and evaluation, with solid health information systems that generate reliable data and support the application of information for improved decision-making and learning at the community, country and international levels [13]. In collaboration with the WHO, it established a new conceptual framework for PC to comprehensively assess its provision and integration into global health systems [13].

### Further evolution of the concept, methodology and graphics

The initial versions of the PC atlases were very comprehensive, capturing a wide array of thematic areas and establishing a narrative rich with data. As the editions of the atlas grew in detail and breadth, with volumes for Europe [14] and Latin America [15] spanning hundreds of pages, the need for a more succinct and comparative format emerged. This development led to the creation of ‘cartographic versions’ of the atlas, which offered a comparative regional perspective and descriptive analysis through infographics, making complex information more accessible and visually digestible.

With time, the methodology of the atlas advanced, aiming to improve comparability across countries. It increasingly focused on the dimensions outlined in the WHO public health framework [16]. The presentation of the information has evolved considerably over the more than 15 years of the project, resulting in 8 publications (7 atlases and a special supplement) corresponding to 4 major world regions: Europe, Latin America, Africa and the Eastern Mediterranean (Table 1). The APCA Atlas for Africa represented a pivotal moment in this evolution, as it adopted a regional consensus process to determine the most effective indicators [17]. This strategy was further solidified by publishing a manual of indicators grounded in a comprehensive literature review, setting the foundation for the latest EAPC European Atlas [18, 19].

**Table 1:** Palliative care atlases developed by the ATLANTES team

Title	Year of publication	Number of countries included	% by WHO region	Number of general maps	Link
EAPC Atlas of Palliative Care in Europe	2007	43	81%	6	-
EAPC Atlas of Palliative Care in Europe 2013 - Cartographic Edition	2013	46	87%	18	DADUN: EAPC Atlas of Palliative Care in Europe 2013 - Cartographic Edition
Atlas de Cuidados Paliativos de Latinoamérica. Edición cartográfica 2013	2013	19	58%^1^	12	DADUN: Atlas de Cuidados Paliativos en Latinoamérica. Edición Cartográfica 2013
Specialisation in Palliative Medicine for Physicians in Europe 2014. A supplement of the EAPC Atlas of Palliative Care in Europe	2014	18	33%	4	DADUN: Specialisation in Palliative Medicine for Physicians in Europe 2014 - A supplement of the EAPC Atlas of Palliative Care in Europe
Atlas of Palliative Care in the WHO-EMR	2017	15	71%	16	DADUN: Atlas of Palliative Care in the Eastern Mediterranean Region
APCA Atlas of Palliative Care in Africa	2017	48	91%^2^	19	DADUN: APCA Atlas of Palliative Care in Africa
EAPC Atlas of Palliative Care in Europe 2019	2019	51	96%	25	DADUN: EAPC Atlas of Palliative Care in Europe 2019
Atlas de cuidados paliativos de Latinoamérica 2020	2021	17	52%^1^	12	DADUN: Atlas de cuidados paliativos de Latinoamérica 2020 (2ª ed.)

^1^ The WHO Americas region comprises a total of 35 countries. However, the percentage calculation was made over 33, excluding Canada and the United States

^2^ The African atlas includes data from 5 countries that are not part of the WHO region, so they have been excluded from the calculation

EAPC (European Association for Palliative Care); ALCP (Latin American Association of Palliative care); APCA (African Association of Palliative care); EMR (Eastern Mediterranean Region)-WHO, and AFRO-(Africa Region)- WHO

The first atlas was commissioned by the European Association for Palliative Care (EAPC) 20 years ago when they were aware of the success of the Spanish PC Directory of Services. They wanted to map the PC development in Europe. The ATLAS project emerged from the imperative to understand and enhance global PC provision. The initial idea evolved through key collaborations with organisations such as EAPC, ALCP (Latin American Association of Palliative Care), APCA (African Association of Palliative Care), EMRO (WHO-Eastern Mediterranean Region Office), and AFRO (WHO-Africa Region Office), and the backing of dedicated funding and institutions. Initially aiming for thoroughness in collected information, the technical team and scientific associations involved realised the need to refine data collection and analysis methods. This manifested in adopting a more robust model focused on the quality and precision of indicators.

Publishing thematic articles has been challenging but has enabled the team to transcend mere descriptive or comparative presentations, providing critical and thematic analysis in areas such as education, services, policies, and research. Comparisons across different editions have revealed trends and the evolution of PC in each region, thus contributing to the continuous improvement of health systems.

The University of Navarra funded the Atlas project; however, the team has also sought additional funding sources. The first European Atlas was supported by an educational grant from a pharmaceutical company and subsequent editions have been co-sponsored by the IAHPC (International Association for Hospice and PC), the University of Glasgow, and the University of Bologna with a contribution for the edition and publication of the monographies. This was not a biomedical research project but an evaluation of PC program implementation phases worldwide, aligning with the Steering Committee on Bioethics of the Council of Europe’s “clinical audit.” Atlases projects have been approved by the University of Navarra and other ethics committees.

The most recent Atlas has addressed PC integration into national health systems, providing an advanced framework for regions with established PC services. The collaborative efforts with scientific associations and the strategic use of conferences for dissemination have been instrumental in this evolutionary process, reflecting on the quality and quantity of health indicators used to gauge PC development. All atlases have, in addition to an introductory section, two major blocks of information. The first analyses the development of PC for the region as a whole (thematic maps), while the second presents the information from a national perspective (country reports). In both sections, the key that has guided the project’s development in graphic design has been providing increasingly complete information in terms of spatial and thematic coverage and more visually and attractively.

**Fig. 1:**
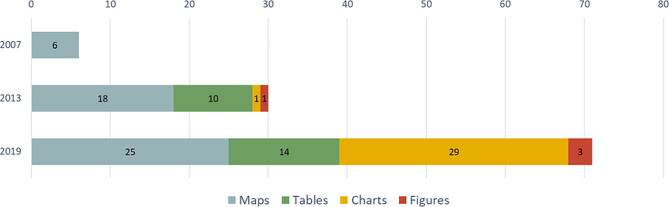
Number and type of graphic resources used in the thematic parts of European Atlases

The latter is well exemplified by the development of the atlases of Europe (Fig. 1). The thematic part, unaffected by the increase in the number of countries presented in each edition, has grown from six graphic elements in 2007 to 30 in 2013 and 71 in 2019. This is a very significant increase, not only in terms of numbers but also in terms of the variety of formats, especially the number of tables and graphs.

**Fig. 2:**
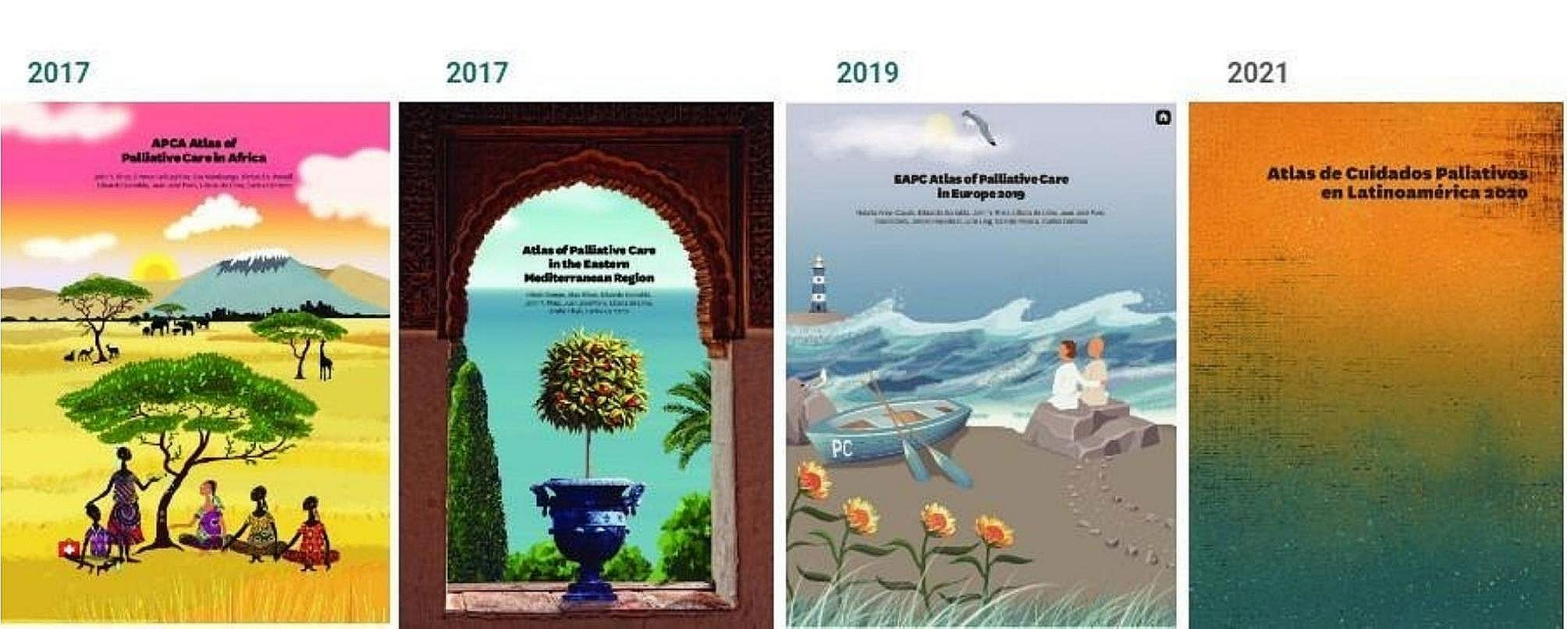
The atlas covers European, Latin American, African and Eastern Mediterranean regions

In general, each atlas has been designed with a box layout, cartographic projections and map scales adapted to the specific area of study, depending on the spatial scope covered. In almost all publications, with the exception of the first atlas and the 2014 supplement, several working scales have been used per atlas, ranging from a double-page map to four maps per page. The graphic design has also evolved. The first European atlas (EAPC, 2007) used different colour schemes for each of the maps dealing with different themes, as well as a set of pictograms that set it apart from all others in terms of design [20]. However, since the 2013 edition of the Atlas of Europe, the map designs have remained quite similar between the different projects, always using sober cartography as a sign of identity, with a base map in soft grey colours, a very simple symbology (avoiding the pictograms of the first atlas) and a common typography. Figure 2 shows the atlas covers of European Latin American, African and Eastern Mediterranean regions. The atlases have been produced using different versions of ESRI software, from ArcView 10.0 to ArcGIS Pro 2.6.

Cartography and infographic design have been an essential part of the Atlases, but behind each Atlas is a broader editorial and communication effort. Its main objective is to communicate the message of PC worldwide. A message that speaks of caring life, human values, professional effort, excellence in the attention, and dignity of seriously ill patients. For that reason, the editing of each monograph is meticulous, with visually attractive and colourful covers, typography and interiors designed in every detail, with introductory sections that talk about the work teams and the methodological rigour of the study, with lists of collaborators by country and inner covers that always feature patients, families, and professionals graphically reminding what is later expressed in tables and maps. This development led to the creation of ‘cartographic versions’ of the atlas, which offered a comparative regional perspective and descriptive mixed analysis through infographics, making complex information more accessible and visually digestible. The Atlases are designed in detail to be a credential of PC, a professional business card used to introduce the concept in ministries, advisory boards, and hospitals. All the ATLASES have maintained the same graphic identity, thus speaking of a cooperative effort that transcends geography and is global.

### Key messages from regional atlases on PC trends

- In 2019, ATLANTES conducted a systematic review to update the PC development and integration analysis across Europe [18]. A previous assessment was carried out in 2013 [21]. It presented 51 country reports highlighting key data on national policies, medicine use, education, and PC service provision. **Although preliminary data on PC integration in different settings are encouraging, disparities between countries and sub-regions remain.**
- The first Atlas of Palliative Care in Latin America, published in 2013, provided regional information from 19 Latin American countries [22]. The second edition, published in 2021 [15], included 17 countries, representing nearly 9% of the global population. The atlas showed that over 3.5 million people in Latin America live in suffering, with over 1 million dying. **The coverage was around 7.6%, with most PC teams operating exclusively in hospitals, with mobile teams being the predominant model.**
- The APCA Atlas of Palliative Care in Africa (2017) [17] analysed PC development in Africa, focusing on the WHO’s Public Health Strategy for integrating PC: policies, medicines, education, and service provision. **It found that Uganda, South Africa, and Kenya have the highest number of specialised hospice and PC services, while 19% have no services.** Opioid consumption per capita is low, with Mauritius, South Africa, Namibia, and Morocco having the highest consumption.
- The Atlas of Palliative Care in the WHO-EMR (2017) [23] was the first of its kind to conduct a systematic descriptive analysis of PC development in the region. PC remains underdeveloped in most countries, necessitating increased training for health professionals to improve symptoms and pain management. **Balancing the need for capacity building with investment in future specialists is challenging, as access to pain medicines remains a pressing issue in low- and middle-income countries.**
- The update of the EMR Atlas is in the publication phase, and new versions for Europe, Africa, Latin America and Asia are still in the scientific production phase.

**Fig. 3:**
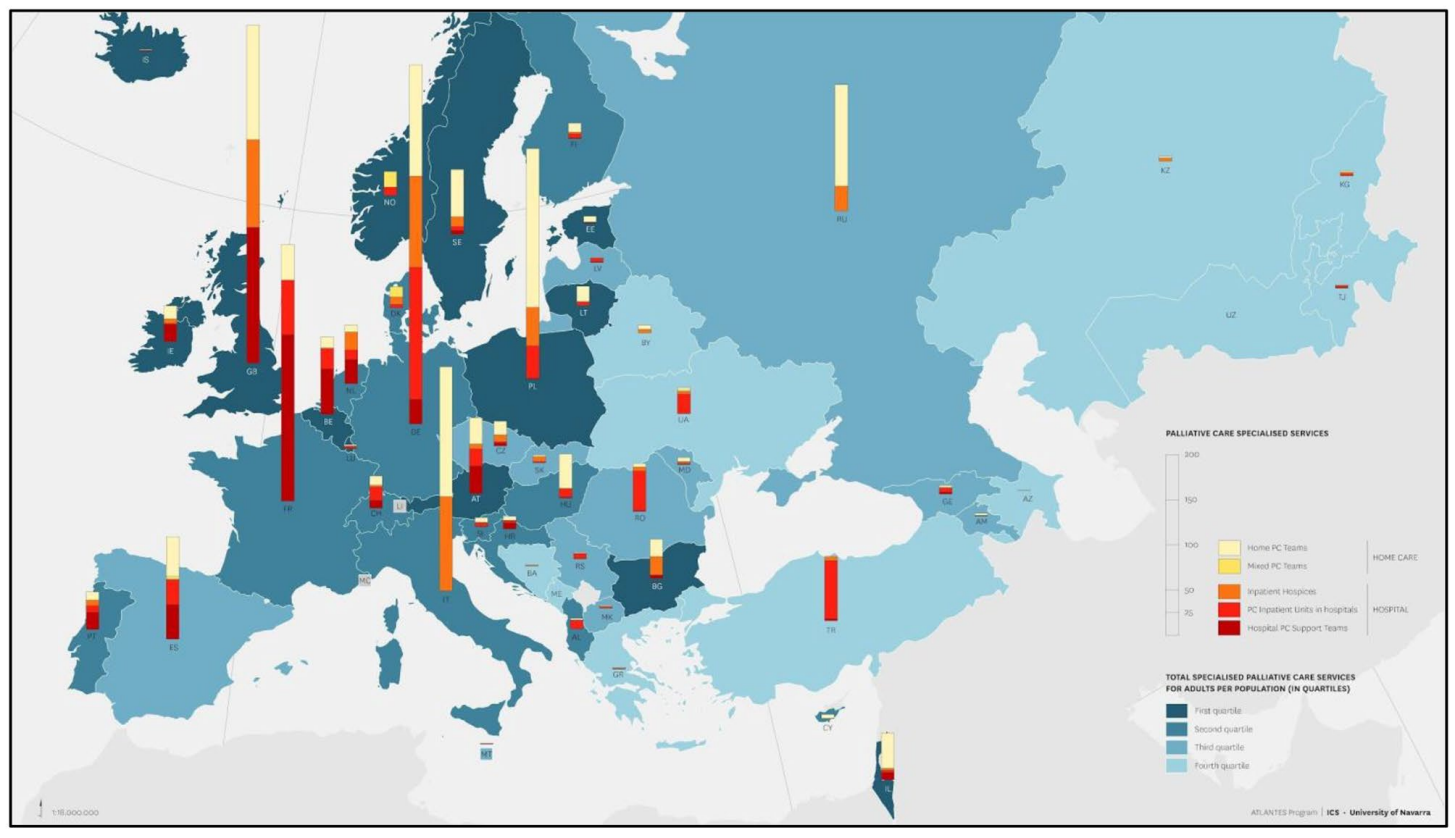
European distribution of palliative care specialised services (with permission of Arias-Casais et al. 2019)

**Fig. 4:**
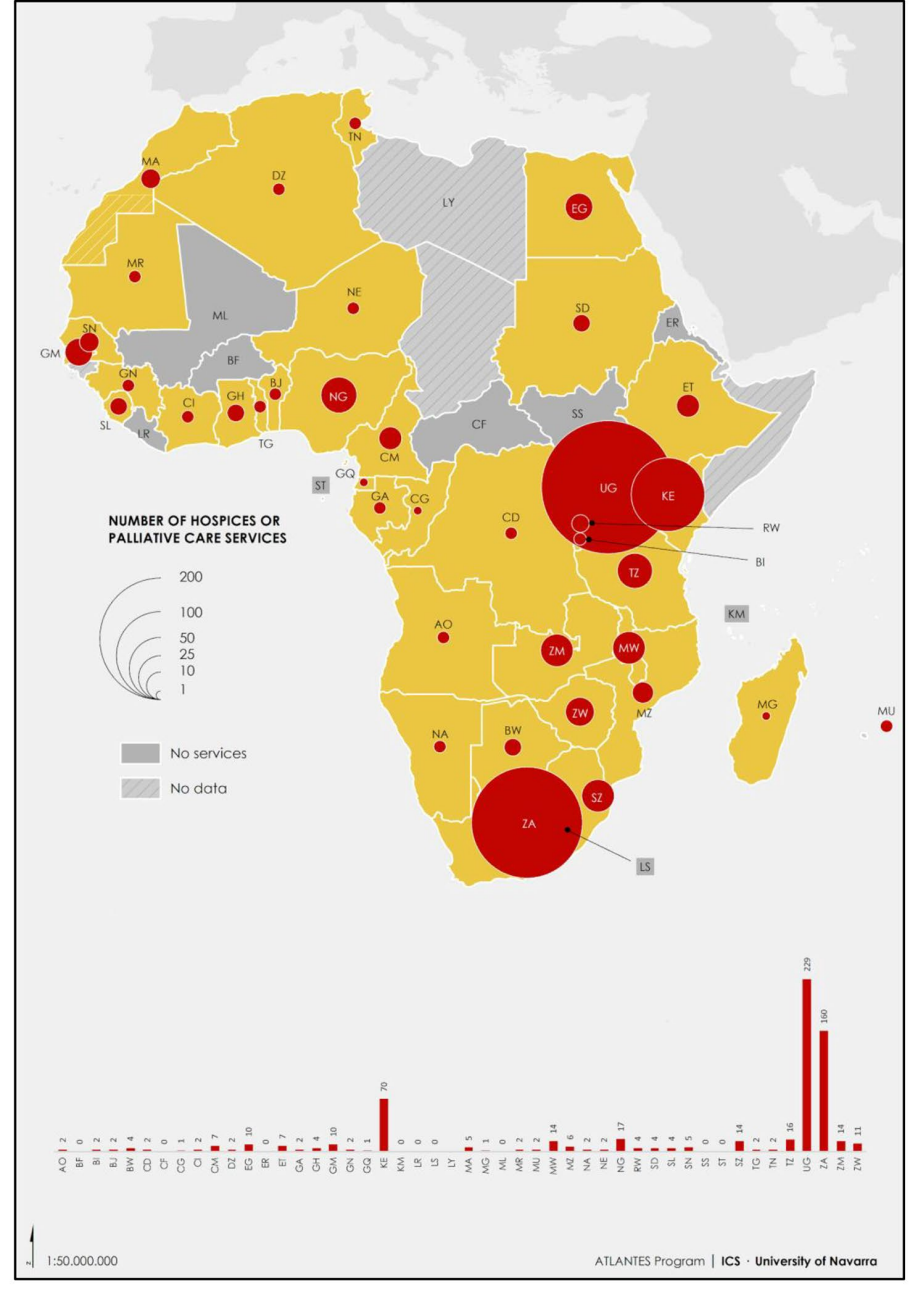
African distribution of palliative care specialised services (with permission of Rhee JL et al., 2017)

The following are examples of maps from Europe (Fig. 3) and the African continent (Fig. 4). In Europe, it is easy to visualise the distribution of specialised PC services and also specialised adult services per population. Similarly, for Africa, the number of hospices or PC services per country.

### Quantitative and qualitative atlases impact

The Atlas project focuses on the quality and precision of indicators, refining data collection and analysis methods to enhance global PC provision. The research team has successfully published thematic articles, critically analysing areas like education, services, policies, and research. Comparing different editions has revealed trends and the evolution of PC, contributing to continuous improvement in health systems. Atlas-type studies have a public health, macro, and comparative perspective, allowing data contextualisation and identifying good examples. Mapping is used to identify areas for improvement and prioritisation. Since the first European atlas in 2005, regions have changed, and PC development indicators and information quality have been refined. The evolutions and impact of this project on PC could be tracked through several official documents citing the atlases and supporting health policies based on these findings [13, 24].

The first EAPC atlas had an inventory of specialised services for the continent. It explored the four dimensions that the WHO envisaged for the integration of PC into health systems (education, services, opioids and policy) [16, 25]. The observatory added a new dimension: professional activity. That atlas obtained information from 43 countries, and its results were presented in national reports [20]. Subsequently, the European Atlas was updated by employing a very extensive double questionnaire (quantitative and qualitative), whose informants were mostly members of national associations. Ninety respondents from 50 countries participated, and the result, in addition to comprehensive country reports, was a cartographic edition presenting the main indicators in a comparative way [26].

The experience of the European mapping edition, replicated in the ALCP Atlas 2013 [27] and a set of secondary analyses of the atlas data [28, 29], allowed us to understand that there were indicators that were better predictors of development than others, i.e. the same could be measured with fewer indicators. Thus, in 2017, the literature was reviewed to understand how PC was measured globally and whether there were groupable indicators. From this study, which resulted in 375 indicators, 40 core indicators were subjected to an international consensus to understand which were globally the most relevant, measurable and feasible [30, 31].

The significant challenges since 2017, as some articles pointed out, were the uneven relevance of indicators according to geographical contexts and the use of experts as sources of information [32, 33]. With help from the APCA, ATLANTES worked to refine the indicators (co-designing them with African leaders) and to refine the informant criteria for the Africa Atlas. In addition to being nominated by APCA, the informants had to be members of national societies and authors of publications with clinical expertise and national knowledge of the PC situation. In addition, their names were validated by other international organisations, and their reports were triangulated with those of other experts in the country. Data were reconciled by consensus and using ad hoc literature reviews in case of conflicting information. The result was the most comprehensive assessment, with 13 indicators for 51 African countries. Following that example, in 2017, the Atlas of the WHO-EMR was published in collaboration with the American University of Beirut (Lebanon) [23].

The African experience (characterised by developing context-relevant indicators and strict information sources) was consolidated in the 2019 European assessment. This also explored the integration of PC at different levels of care (primary care), other non-cancer pathologies (cardiology), in the paediatric population, by other health care providers (volunteers) and in different care settings (nursing homes), to name a few. Each area of research had indicators that agreed with the EAPC working groups and self-reporters. In total, more than 400 experts from 54 countries mapped the situation in Europe [18] and allowed for multiple secondary analyses on paediatrics, coverage, integration, and policies) [19, 34].

The Observatory collaborated with the Atlas of Latin America 2020, whose main novelty - and difficulty - was incorporating official sources and subsequent disagreements with independent sources [15]. Data were collected from 50 experts from 17 countries, highlighting the insufficient and heterogeneous reality of Latin America and the lack of official records on PC.

With ATLANTES as its collaborating centre, the WHO has set three visions for the global assessment and monitoring of PC since 2021: reducing indicators, renewing palliative development concepts, and framing PC as an essential part of the health system [13, 35]. The first exercise under these objectives was the EMR PC Status Report [36].

Today, the Observatory has developed a global assessment using 14 WHO indicators tested in Benin, Morocco, and Uruguay [37]. It employs a network of independent quality reporters and offers assistance in in-depth country assessments. Informants are trained in an official course at the University of Navarra to enhance information quality. The country report is validated through a literature search and published on the Observatory’s digital dashboard.

These atlases presented the most relevant information on PC development in a clear, accessible and easily interpretable way for professionals, policymakers and the general public. In addition, in 2022, ATLANTES conducted an international survey to identify key stakeholders and assess dissemination channels. It provided insights into effective communication strategies for maximum reach and relevance, supporting its mission to enhance PC global development [38]. Scientific articles and atlases were preferred products to reach academics and general practitioners.

These findings may be aligned with making PC part of the essential portfolio of health services provided to the whole population, globally, whatever the national context, particularly in countries where it is almost non-existent, by increasing access to existing knowledge.

### Current and future vision of palliative care atlases

The ATLANTES Global Observatory has navigated a complex landscape of PC research, facing numerous challenges, learning valuable lessons, and achieving significant milestones. To date, the observatory has published a series of influential PC Atlases, all accessible through the DADUN repository (Table 1). A search on PubMed reveals more than 20 papers from authors of the ATLANTES groups and collaborators or colleagues that have contributed to advancing PC development studies.

One of the primary challenges faced was the critical need for stakeholder engagement with the findings of PC research. To address this, ATLANTES conducted an international e-survey among Observatory collaborators to explore key audiences, the best ways to reach them, and priority activities. The study highlighted the importance of prioritising policymakers and healthcare practitioners as key stakeholders in promoting PC and driving its global development and integration into healthcare systems. Innovative web tools and social networks were identified as effective means to extend the outreach efforts beyond the PC community [38].

The ATLANTES publications have been cited extensively and utilised as advocacy tools within international institutions and ministries of health. They have played an instrumental role in informing policy and guiding the global expansion and improvement of PC services. For instance, the EAPC Atlas (2013) achieved 434 citations, the Atlas of PC in the EMR (2017) achieved 136 citations, the APCA Atlas of PC in Africa (2017) 118 citations, and the Latin American Atlas (2021) 45 citations. Furthermore, the PC Atlases have become a mark of excellence for professionals in the field, allowing them to demonstrate their expertise and commitment to their colleagues and in multidisciplinary healthcare settings.

ATLANTES’ work, particularly the “Brief Manual on Health Indicators Monitoring Global Palliative Care Development“ [30], has been foundational in offering a more comprehensive assessment of PC provision. This manual and the atlases garnered the attention of the WHO, which led to ATLANTES coordinating an international consensus to establish a new conceptual framework for developing PC, defining core and strategic indicators in each dimension [13].

**Fig. 5:**
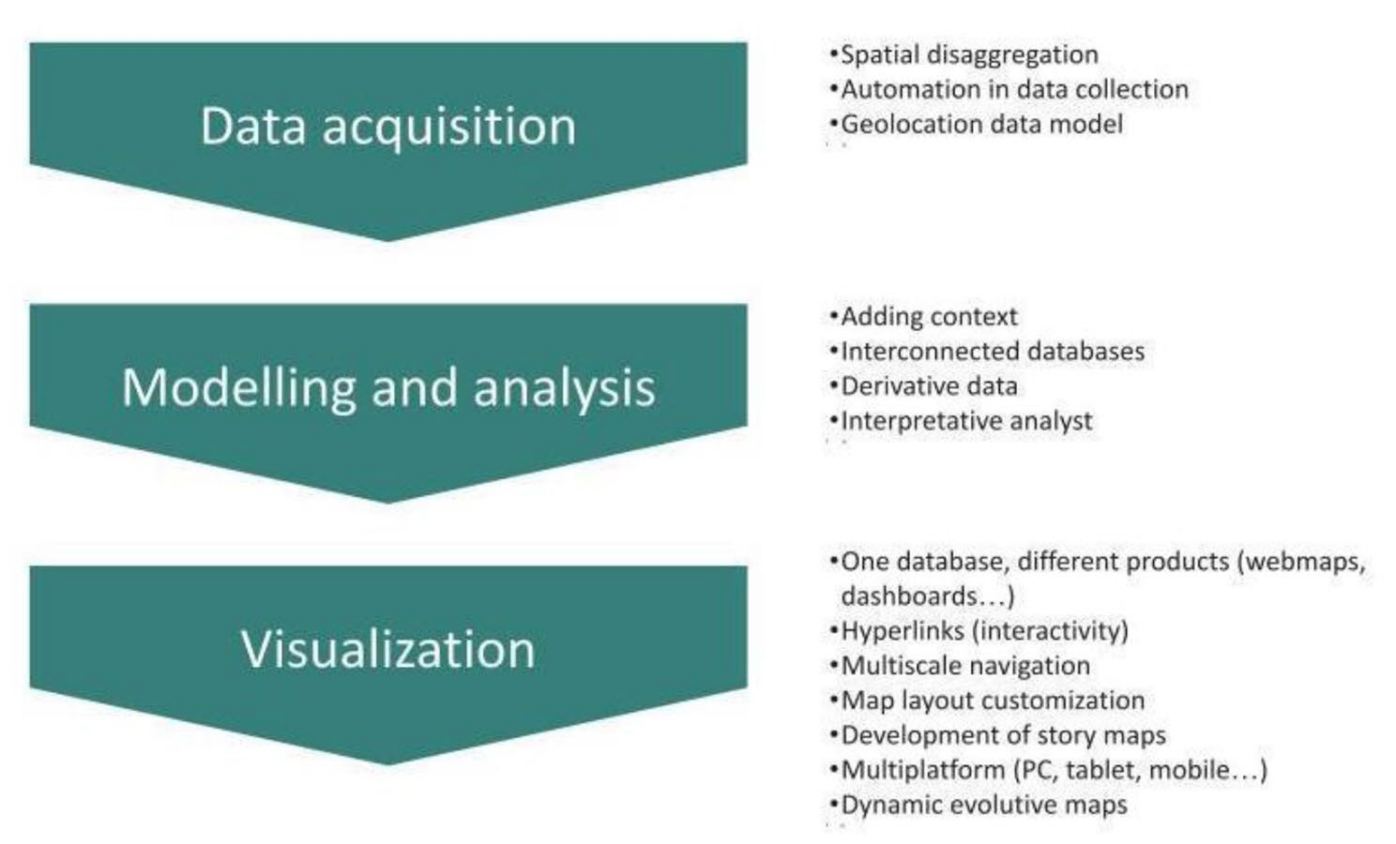
ATLANTES conceptual evolution of data acquisition, data modelling, analysis, and visualisation

The transition from being an Observatory to being designated as the WHO Collaborating Centre for the Global Monitoring of PC Development marked a significant evolution. ATLANTES is now tasked with combining expert and official sources, presenting findings in real-time through an interactive dashboard already impressing stakeholders. This dashboard will be continuously updated with fresh and validated information, ensuring that the data remains relevant and reflective of the current state of the global PC. Figure 5 shows the innovative and dynamic conceptual evolution of data acquisition, data modelling, analysis, and visualisation face to the updated technologies.

The peer-reviewed publications, though initially challenging due to editorial misunderstandings distinguishing them from monographs, have subsequently been crucial in lending scientific credibility to the project. As we embark on this new phase, we aim to maintain the rigour and depth that have characterised our work while embracing the opportunities technology offers to broaden our impact and ensure that our contributions to PC are accessible and actionable.

The Global Observatory of PC emphasises the importance of building networks of collaborators at all levels to address PC’s diverse needs and contexts across different regions. International organisations like the IAHPC and the World Hospice Palliative Care Alliance (WHPA) sponsored atlases facilitated by regional organisations. The network of over 700 key informants from various mapping projects has contributed to the success of ATLANTES, providing data, co-designing studies, fine-tuning indicators, and interpreting findings. These networks lead the work, and the Observatory must meet and learn from their expectations. The ATLANTES collaborators have highlighted the importance of prioritising policymakers and healthcare practitioners as key stakeholders in advancing palliative care. Social networks and website tools can further disseminate the Global Observatory data. Working with these international networks can significantly increase the observatory’s research effects [38].

**Fig. 6:**
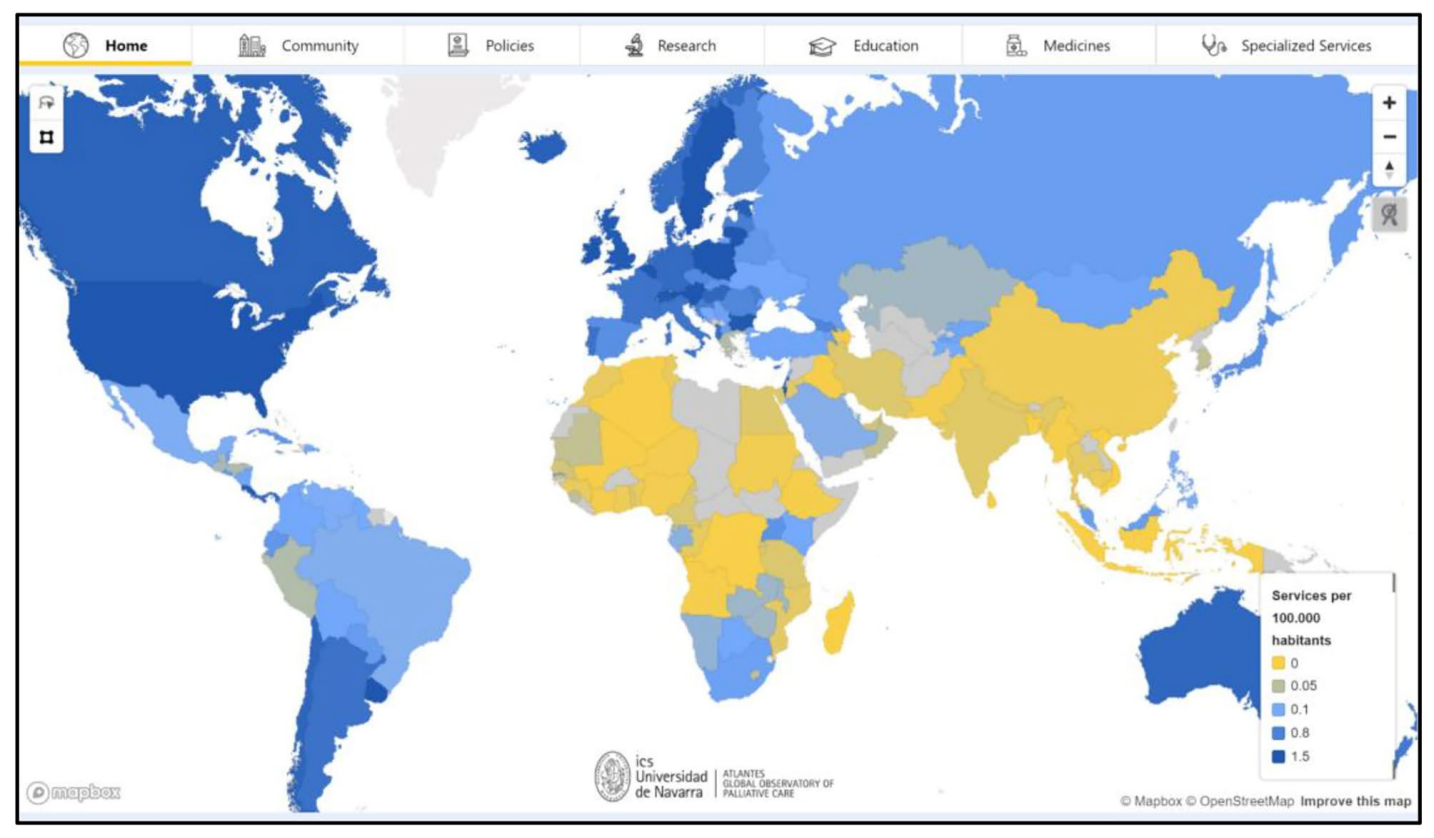
Interactive dashboard of global palliative care development

ATLANTES has shifted from static maps and infographics to big data interactive visualisation web tools to democratise access to evidence-based PC data (Fig. 6).

The initial step involved consolidating information from regional atlases, World Maps of PC, and socio-economic databases. The goal was to make data accessible, self-explanatory, and visually attractive. PowerBI was chosen as the primary engine for creating dashboards based on criteria and specialised training. Two dashboard types were designed: a cross-country comparable approach and an in-depth country infographic style. The first dashboard features maps and graphs, allowing users to interact with development dimensions in six tabs: empowered people and communities, health policies, research, education, use of essential medicines, and integrated services-related data. The unified graphic identity between the two dashboard types was sought to enhance presentation and user experience. Real-time information visualisation is pursued, ensuring the latest quality data is displayed. Maintenance, periodic updates, and leveraging the Observatory’s informant network are crucial for the platform’s impact.

### Limitations

There are limitations to our work. Firstly, the data is provided by experts, and the criteria for their selection and accreditation are not always verifiable. To minimise this likely bias, we required the agreement of national and regional CP associations. In addition, training and collaboration with the experts allowed us to minimise data variability. On the other hand, the quality of the information was not always ratified or corrected by official sources in the Ministries of Health or other government agencies. Dynamic dashboards allow for much more up-to-date data, while the atlases can be considered a cross-sectional assessment. Finally, the indicators used for global assessments may not be the most appropriate for specific country situations.

## Conclusion

For several years, ATLANTES has produced comprehensive regional CP atlases [14, 15, 17, 23] and published numerous articles that summarise and analyse the key findings of each report [19, 28, 39–41] and infographics. These Atlases provided information to key stakeholders like healthcare professionals, ministries of health, and nongovernmental organisations to advocate for enhanced PC access in several regions. Consolidating an open-access web mapping tool will provide all available information from official and published sources with high-quality data. This design will allow stakeholders to perform ‘’tailored’’ searches at national, regional and global levels. Leveraging existing knowledge is essential to bridge the gap and ensure equitable care, especially in countries with limited access to PC. It may not be in vain to increase efforts in policy briefing and dissemination; policymakers are an essential first step in organising and delivering services at the national level.

## Data Availability

all data from the Atlases and webtool are available open access at the ATLANTES website, University of Navarra https://www.unav.edu/web/atlantes-global-observatory-of-palliative-care/monitoring.
